# Physiology, imaging and proteomics of non-ventilated vs. non-perfused lung injury: an experimental study

**DOI:** 10.1186/s40635-025-00772-6

**Published:** 2025-06-23

**Authors:** Anna Damia, Ines Marongiu, Elena Spinelli, Francesco Damarco, Clarissa Uslenghi, Giovanni Lorenzo Rumi, Michele Battistin, Caterina Lonati, Alessandra Maria Storaci, Gianluca Lopez, Maria Rosaria De Filippo, Fabiana Madotto, Cristina Banfi, Alice Mallia, Lorenzo Rosso, Valentina Vaira, Tommaso Mauri

**Affiliations:** 1 Department of Pathophysiology and Transplantation, University of Milan, Milan, Italy; 2 Department of Anesthesia, Critical Care and Emergency, Fondazione IRCCS Ca’ Granda Ospedale Maggiore Policlinico, Milan, Italy; 3 Division of Thoracic Surgery and Lung Transplantation, Fondazione IRCCS Ca’ Granda Ospedale Maggiore Policlinico, Milan, Italy; 4 Center for Preclinical Research, Fondazione IRCCS Ca’ Granda, Ospedale Maggiore Policlinico, Milan, Italy; 5 Division of Pathology, Fondazione IRCCS Ca’ Granda Ospedale Maggiore Policlinico, Milan, Italy; 6 Department of Biomedical Surgical and Dental Sciences, University of Milan, Milan, Italy; 7 Centro Cardiologico Monzino I.R.C.C.S, Milan, Italy

**Keywords:** Ventilation, Perfusion, Lung injury, Inflammation, Imaging, Proteomics

## Abstract

**Background:**

The exclusion of one lung from ventilation or pulmonary artery perfusion triggers pathological mechanisms that can lead to lung injury. Although the final effect is similar for both insults, the underlying mechanisms may differ. Primary aim of this study was to compare severity of lung injury between non-ventilated (NVLI) and non-perfused (NPLI) lung injury. Secondary aims were to compare physiologic, imaging and proteomic signatures of NVLI vs NPLI.

**Methods:**

Sedated and paralyzed healthy female piglets (weight = 36 ± 5 kg) were mechanically ventilated for 24 h after left pulmonary artery ligation (NPLI group, *n* = 11) or exclusion from ventilation of the left lung (NVLI group, *n* = 10). Physiological data including electrical impedance tomography imaging of regional ventilation and perfusion were collected. Histological scoring was performed blindly as well as proteomic analysis of broncho-alveolar lavage (BAL) fluids and lung tissue samples at the end of the experiment.

**Results:**

The left lung of both groups received similarly low fraction (< 20%) of blood flow. The left side of the NPLI group was characterized by ventilation distributed only to the dead space and high ventilation/perfusion compartments, while the left lung of the NVLI group was characterized by perfusion only to the shunt compartment. The left lung of the NVLI group showed severe pulmonary vascular dysfunction (pulmonary vascular resistance > 2000 dyne/s/cm^−5^), while the left lung of the NPLI group was ventilated with raising inspiratory stress (driving pressure > 20 cmH_2_O at the end of the experiment and progressive decline in left lung compliance). The histologic lung injury score was higher for the left lung of the NVLI group compared to the left lung of the NPLI (left histological score: 10.3 ± 2.0 vs 6.4 ± 1.6, *p* < 0.0001), and pro-inflammatory alveolar cytokines were similarly more expressed in the left lung of the NVLI versus NPLI group (IL-1β: 418 ± 416 vs 53 ± 71, *p* < 0.001; IL-6: 406 ± 455 vs 99 ± 93, *p* = 0.036). Proteomic analysis showed signature specific for the two injuries, with two proteins, namely PRDX5 and DCTN1, being upregulated in NVLI left lung compared with the left NPLI lung. The right lung developed injury only in the NVLI group (right histological score: 5.5 ± 1.9 vs 3.0 ± 0.7, *p* < 0.001).

**Conclusions:**

Lung injury is more severe in terms of lung histological score in the collapsed lung of the NVLI group and involves also contralateral areas. At the mechanistic level, NVLI has specific physiologic mechanisms like vascular dysfunction and inflammation and presents unique proteomic profile in comparison to NPLI.

**Supplementary Information:**

The online version contains supplementary material available at 10.1186/s40635-025-00772-6.

## Background

The lungs of patients with acute respiratory failure are characterized by presence of regional ventilation/perfusion (V/Q) mismatch, where ventilation and/or perfusion are excessive, defective or completely lacking. Derangements of pulmonary V/Q matching have long been known to cause impairment of oxygenation and CO_2_ elimination. Subsequent clinical studies showed that the severity of hypoxia and higher inefficiency in CO_2_ clearance are very good prognostic markers [1, 2]. Thus, V/Q mismatch causing alteration of gas exchange could be seen as a hallmark of the severity of patients with acute respiratory failure.

Nonetheless, the correlation between the different types of V/Q mismatch and respiratory failure is complex and difficult to dissect in the clinical setting for three main reasons:

- V/Q mismatch could cause lung tissue injury within the poorly matched region: loss of ventilation inducing collapse and wasted ventilation of non-perfused regions trigger local injurious mechanisms, including hypocapnia, hypoperfusion, inflammation and apoptosis [3, 4].
- Regions of V/Q mismatch could also induce lung tissue damage in the remote more normally ventilated and perfused lung regions: for example, dead space compensation shifts ventilation and perfusion and could promote ventilation-induced lung injury (VILI) to the residual lung [5].
- Classical bedside measures of the different types of V/Q mismatch (i.e., calculated shunt and physiological dead space) are functional measurements which, often, do not reflect the real extent of the mismatched regions: shunt can underestimate the amount of collapse [6], dead space can be affected by shunt, and poor hemodynamics can alter both [1].

The experimental setting could be ideal to differentiate lung injury caused by presence of non-ventilated vs. non-perfused lung regions in terms of physiological and imaging derangements, local and distal severity and proteomic profiling. In the present study, we characterized two experimental models of complete V/Q mismatch of the left lung by exclusion from ventilation (non-ventilated lung injury, NVLI) vs. unilateral pulmonary artery ligation (non-perfused lung injury, NPLI) in healthy female pigs.

Our hypotheses were: that physiologic derangements could overlap only in part and with different intensities; that severity of lung injury could thus be different, both at the local and remote level; and, finally, that proteomic profile could differ following activation of specific pathways.

Primary aim of this study was to compare severity of histological damage between non-ventilated (NVLI) and non-perfused (NPLI) lung injury in the left lung. Secondarily, we aimed to profile physiologic, imaging and proteomic signatures of NVLI vs NPLI, and to compare severity of injury in the right lung.

## Methods

The study was approved by the Italian Ministry of Health, Rome, Italy (Auth. No. 246/2022-PR, Protocol No. 568 EB.34 and No. 190/2023 Protocol No. 568 EB.41) and conducted according to the European Directive 2010/63/EU on the protection of animals for scientific studies and the Italian decree 26/2014. Approval by the Institutional Committee for Animal Care of the Maggiore Policlinico Hospital of Milan, Italy, was obtained before starting the experiments. All procedures were conducted in full compliance with the ARRIVE guidelines.

### Animal preparation

Twenty-one healthy female pigs—Sus scrofa domesticus—(36 ± 5 kg) were anesthetized, tracheostomized, and instrumented with pulmonary artery, carotid artery, and central jugular vein catheters, according to previously published works by our group [4, 5] (see Online Supplement, section “Supplementary methods”, for details). A left-sided double-lumen endobronchial tube of 37 Fr was used in animals from the NVLI group, while standard endotracheal tube was used in the non-perfused lung injury NPLI animals. Pulsoxymetry, heart rate, invasive arterial pressure and pulmonary artery pressure were continuously monitored. End-tidal CO_2_ and physiological dead space were measured through volumetric capnography connected to the ventilator circuit. Electrical Impedance Tomography (EIT) belt was positioned at mid-chest position and esophageal pressure catheter was advanced in the distal third of the esophagus; both were connected to their monitors and properly calibrated.

### Study protocol

After check for healthy baseline condition, animals were allocated to the following study protocols using a computer-generated sequence:

- NVLI group (*n* = 10) underwent exclusion from ventilation of the left lung by inflation of both double-lumen tube cuffs and disconnection of the left side.
- NPLI group (*n* = 11) underwent surgical ligation of the left pulmonary artery through small left thoracotomy, as previously described [5].

Both groups received controlled mechanical ventilation in supine position for 24 h with the same settings: volume controlled mode, tidal volume (Vt) of 15 ml/kg, positive end-expiratory pressure (PEEP) 1 cmH_2_O (i.e., the minimum to maintain adequate pneumatic performance of the ventilator), respiratory rate 15 bpm, I:E 1:2 and FiO_2_ 0.5 (which was increased in case of desaturation). Six animals were used as a control group (see the Online Supplement, Control group section) but were not included in the analysis.

Fluids were administrated by prespecified protocol, targeting stable arterial blood pressure and neutral fluid balance. Additional description of the study protocol can be found in the Online Supplement.

### Study measures

The following physiological data were recorded after 2, 6, 12, 18 and 24 h (T2, T6, T12, T18, T24) from start of study protocol: EIT imaging data of ventilation and perfusion; arterial and mixed venous blood gas analyses; physiological dead space and end-tidal CO_2_ by volumetric capnography; systemic and pulmonary hemodynamics (systolic, diastolic and mean pulmonary artery pressures, pulmonary vascular resistance and pulmonary artery compliance); respiratory mechanics including plateau pressure, driving pressure, respiratory system compliance and transpulmonary pressure by brief inspiratory and expiratory occlusions.

EIT data were recorded at 50 Hz and stored for offline analysis. EIT ventilation and perfusion maps were obtained by dedicated software. EIT perfusion maps were derived from offline analysis of the time-impedance curve obtained by first pass of a 10 ml-bolus of 5% saline solution injected during an end-inspiratory occlusion, as previously described [7]. We measured the regional distribution of ventilation and perfusion between the two lungs and quantified the regional fraction of ventilation and perfusion reaching each compartment of V/Q mismatch (dead space, high V/Q, normal V/Q, low V/Q and shunt). We considered as non-ventilated and non-perfused, respectively, those pixels whose values laid below the 20% of the maximum value within the map.

After collection of the last physiological measures, at T24, we performed right and left side broncho-alveolar lavage (BAL) with 30 ml of 0.9% NaCl solution for each side. BAL fluids were aspirated, centrifuged at 2000 rpm for 15 min and the supernatants promptly stored at − 80 °C for subsequent ELISA and proteomic analyses. Then, animals were euthanized under deep general anesthesia by central injection of a bolus of 40 mEq KCl, and 6 tissue samples from each lung (2 × basal, middle, apex) were collected and fixed for histological evaluation or frozen at -80 °C for proteomic analyses (middle region sample).

### Inflammatory biomarkers and proteomics

BAL fluids were assayed by ELISA commercially available kits to quantify concentrations of acute inflammatory mediators (IL-6 and IL-1β) [8], following the manufacturer’s instruction.

Proteomic analysis was performed on 5 right and 5 left lung tissue homogenates (middle lung region) by Olink technology (Target 96 Immune response panel, Olink Proteomics AB, Uppsala, Sweden), as previously described [9].

*Severity of lung injury.* Blinded expert pathologist assessed the severity of injury in each lung by validated composite histological score, as the sum of 10 sub-items ranging from 0 to 3, as previously described [5]. Values of the 3 samples from each side were averaged to obtain the representative left and right lung histological injury scores (total score range: 0–30 for each lung).

### Sample size

Difference in the histological score of the left lung between the NVLI vs. NPLI groups was designated as the primary endpoint. Sample size was comparable to previous animal studies on similar topic [10]. We also performed a power analysis, based on previous studies [5, 11], hypothesizing left side histological scores of 10.0 ± 3.0 for the NVLI group and 6.0 ± 3.0 for the NPLI group. To achieve a power of 0.8 with an alpha 0.05, the minimum required sample size was 18 animals (9 per group). One or two additional animals per group were included (total *n* = 21) to account for potential dropouts, in order to maintain statistical validity while adhering to the principle of reduction. The inclusion of a small number of additional animals was based on prior experience with similar models, which showed dropout rates of approximately 10–15%. Also, the numerosity was necessary for the pilot exploratory nature of the study.

### Statistical analysis

Data are shown as mean ± standard error or median with interquartile range, as appropriate. Data between groups were compared using unpaired t-test or Mann–Whitney U test. Longitudinal data along the study timepoints were analyzed using mixed-effect model for repeated measures followed by Sidak’s post hoc test, considering groups and time as independent factors and including the interaction between groups and time. A *p*-value < 0.05 indicated statistical significance. Analyses were performed using GraphPad Prism 9 (GraphPad Software, San Diego, CA).

For the proteomic approach, Olink data normalization and standardization was performed as recommended by the manufacturer and NPX values were calculated. Analyses on proteomic data were performed using R version 4.3.2 (R Foundation for Statistical Computing, Wien, Austria) and the R Package “OlinkAnalyze” was used to identify differentially expressed proteins. *P*-values were calculated using the Welch 2-sample *t*-test and corrected for multiple testing with the Benjamini–Hochberg method. Principal component analysis (PCA) was performed to detect the genes that contribute most significantly to the four directions in the PCA plot. Heatmaps and volcano plots were produced using the R package “ggplot2”. Significantly upregulated proteins (*p* < 0.05 and log2 fold change > 0.5) were imported into the Reactome pathway browser, and the overrepresentation analysis tool with Voronoi pathway visualization was used.

## Results

### Histological injury score and inflammation of the left lung

At the end of the experiment, left lungs of both NPLI and NVLI groups appear smaller with areas of consolidation (Online Fig. S1).

**Fig. 1:**
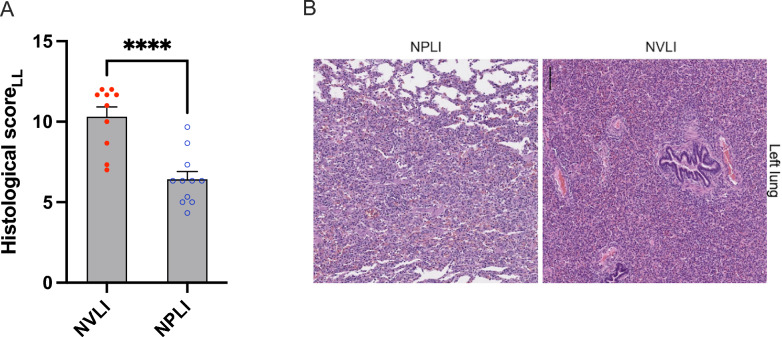
Left lung injury histological score. Lung histological score **A** showed significantly higher values in NVLI vs. NPLI group in the left lung. Representative microphotographs (H&E, scale bar: 300 μm) for the left lung (**B**) of the NPLI (left) and NVLI (right) group are shown. Data are expressed as scatter plot with bars and error bars (mean ± SEM). Statistical analysis is performed by unpaired *t*-test or Mann–Whitney *U* test, as appropriated, p-values are reported in the graphs. ***p < 0.001, ***p < 0.0001. LL, left lung; NVLI, non-ventilated lung injury; NPLI, non-perfused lung injury

**Fig. 2:**
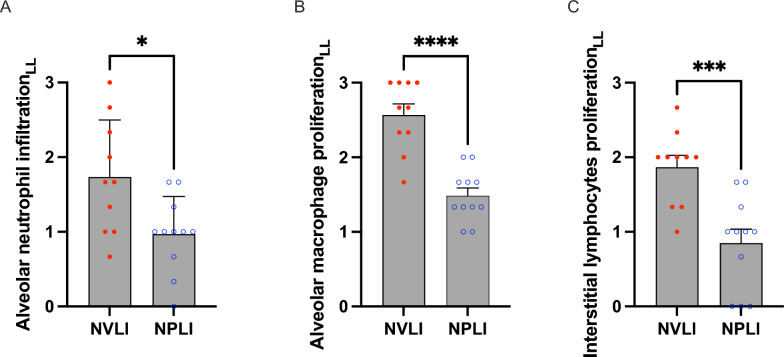
Left lung histological injury score sub-items. The left lung of the NVLI group showed higher alveolar neutrophil infiltration (**A**), macrophage (**B**) and lymphocytes (**C**) proliferation compared to the left lung of the NVLI group. Data are expressed as scatter plot with bars and error bars (mean ± SEM). Statistical analysis is performed by unpaired *t*-test or Mann–Whitney *U* test, as appropriated, p-values are reported in the graphs. *p < 0.05, ***p < 0.001, ****p < 0.0001. *LL, left lung; NVLI, non-ventilated lung injury; NPLI, non-perfused lung injury*

Histological injury of the left lung was higher in the NVLI vs. NPLI group (left histological score: 10.3 ± 2.0 vs 6.4 ± 1.6, *p* < 0.0001, Fig. 1A), suggesting more intense detrimental mechanisms of injury when ventilation is halted as compared to the block of perfusion. Representative histological images of the left lung in the two study groups showed extensive alveolar collapse and more intense cells recruitment/proliferation for the NVLI group (Fig. 1B, right panel). Analysis of histological score sub-items showed more intense inflammatory reaction in the left lung of the NVLI group, as testified by larger alveolar neutrophil infiltration (1.7 ± 0.8 vs 1.0 ± 0.5, *p* = 0.014), and proliferation of macrophages (2.6 ± 0.5 vs 1.5 ± 0.3, *p* < 0.0001) and lymphocytes (1.8 ± 0.5 vs 0.8 ± 0.6, *p* < 0.001) (Fig. 2 A-C). As a reference, histological score for the Control group was low (data reported in Supplementary Table 2).

**Fig. 3:**
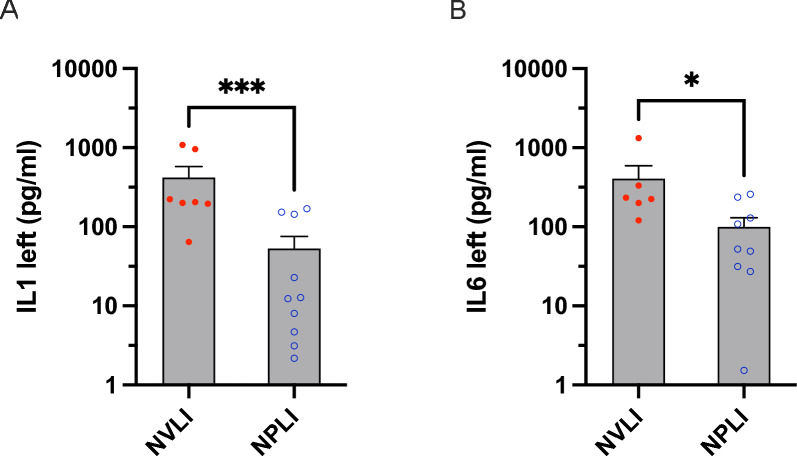
Cytokines concentration from the left BAL fluid. Alveolar concentration of IL-1β and IL-6 was measured in the left lung (**A** and **B**, respectively) of each study group. Data are expressed as scatter plot with bars (mean ± SEM) where each sample is a dot. Statistical analysis is performed by unpaired *t*-test or Mann–Whitney *U* test, as appropriated. *p < 0.05, ***p < 0.001, ****p < 0.0001. NVLI, non-ventilated lung injury; NPLI, non-perfused lung injury

Cytokines levels in the BAL fluid collected from the left lung confirmed results of the histological scores. Higher levels of the acute phase inflammatory cytokines IL-1β and IL-6 in the NVLI vs. the NPLI group (IL-1β: 418 ± 416 vs 53 ± 71, *p* < 0.001; IL-6: 406 ± 455 vs 99 ± 93, *p* = 0.036; Fig. 3A, B) indicate more severe regional inflammation and lung injury.

### EIT imaging: distribution of ventilation and perfusion

**Fig. 4:**
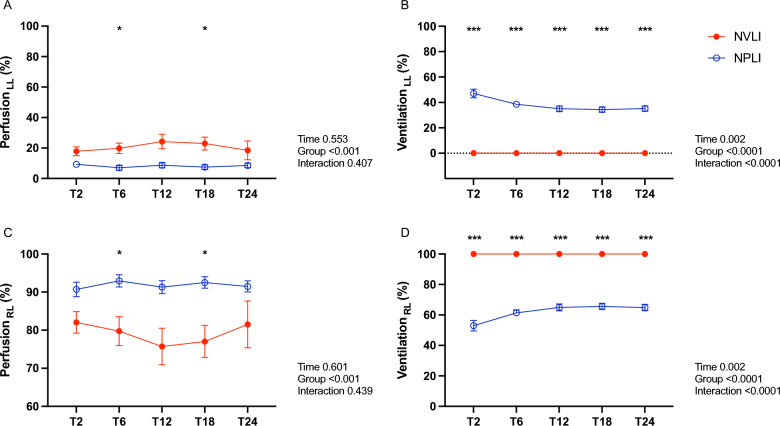
Fraction of pulmonary perfusion and ventilation reaching each lung. EIT-based fraction of pulmonary perfusion and ventilation reaching the left (**A** and **B**) and the right (**C** and **D**) lung in the two study groups. Data are expressed as mean ± SEM. Comparisons are obtained with Mixed-effect analysis for repeated measures followed by Sidak’s post hoc test with groups and time as independent factors and group x time interaction. P-values are reported in the graphs. *p < 0.05, ***p < 0.001. LL, left lung; RL, right lung; NVLI, non-ventilated lung injury; NPLI, non-perfused lung injury

The fraction of blood flow reaching the left lung was low (< 20%) in both study groups (Fig. 4A): this suggests intense local hypoxic pulmonary vasoconstriction (HPV) in the NVLI group and partial compensatory flow from the bronchial arteries for the NPLI group. The left lung of the NVLI group was excluded from ventilation for the whole experiment (Fig. 4B), causing extensive alveolar collapse (see histological images, Fig. 1B). In the NPLI group, the fraction of tidal volume reaching the left lung was reduced to around 40%, probably due to local hypocapnic bronchoconstriction [12] (Fig. 4B).

### Physiologic features of lung injury

*Local V/Q mismatch.* Both NPLI and NVLI groups were characterized by extreme V/Q mismatch in the left lung, albeit with profound differences. In the NPLI group, left regional ventilation was distributed mostly to dead space (Fig. 5.1A) and, to lesser extent, to the high V/Q compartment (Fig. 5.1B), suggesting diffuse alveolar hypocapnia. Left lung perfusion by bronchial circulation mainly flowed through high V/Q compartment (Fig. 5.2A) and shunt compartment (Fig. 5.2D) (attesting development of regional lung edema/collapse), with less than 20% reaching the normal V/Q compartment (Fig. 2.2B).

**Fig. 5:**
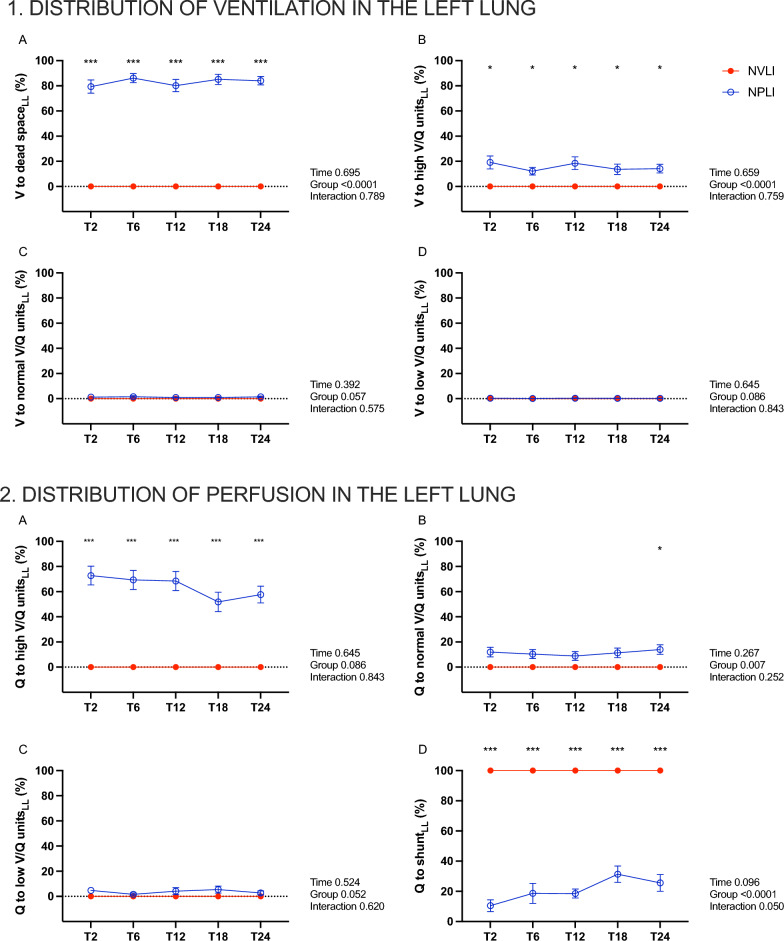
Distribution of ventilation and perfusion to the different V/Q compartments in the left lung. Panel 1: EIT-based fraction of ventilation reaching dead space (**A**), high V/Q (**B**) normal V/Q (**C**) and low V/Q (**D**) compartments in the left lung. Panel 2: EIT-based fraction of perfusion reaching the high V/Q (**A**), normal V/Q (**B**), low V/Q (**C**) and shunt (**D**) compartments in the left lung. Data are expressed as mean ± standard error. For each dependent variable, mixed-effect model for repeated measures was performed to detect significant differences between groups over time (Sidak’s post hoc test). P-values are reported in the graphs. *p < 0.05, ***p < 0.001. V, ventilation; Q, perfusion; V/Q, ventilation to perfusion; LL, left lung; NVLI, non-ventilated lung injury; NPLI, non-perfused lung injury

Absence of ventilation to the left side of NVLI group determined 100% of regional perfusion reaching the shunt compartment (Fig. 5.2D).

**Fig. 6:**
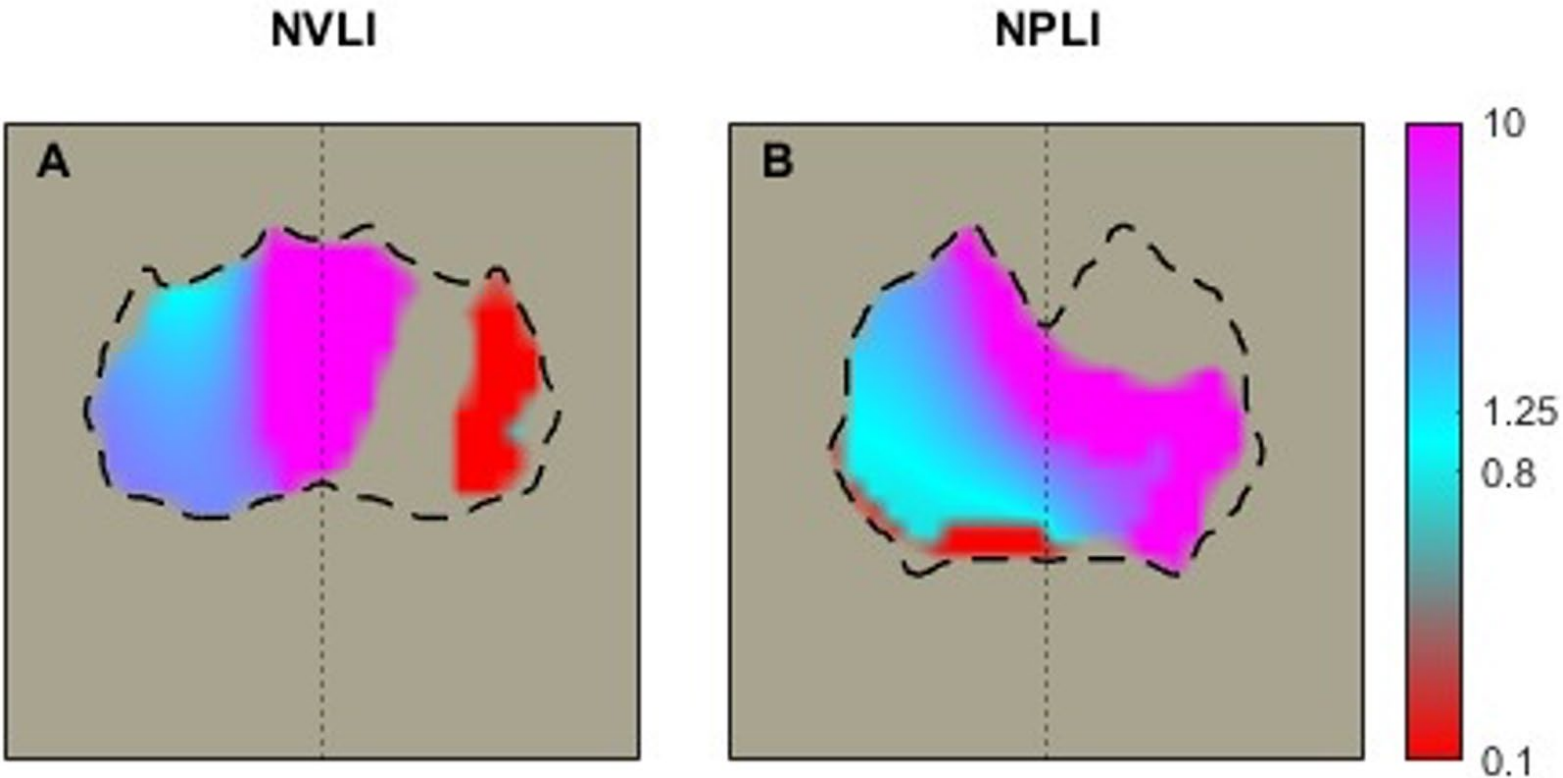
EIT images for V/Q mismatch. Representative images of V/Q mismatch in the NVLI group (**A**) and the NPLI group (**B**). The scale grades from non-ventilated perfused units (shunt) in red to non-perfused ventilated units (dead space) in purple. The normally ventilated and perfused units are represented in light blue. NVLI, non-ventilated lung injury; NPLI, non-perfused lung injury

Figure 6 shows representative EIT images of ventilation, perfusion and V/Q mismatch in the 2 study groups.

#### Accuracy of clinical bedside measures of V/Q mismatch

**Fig. 7:**
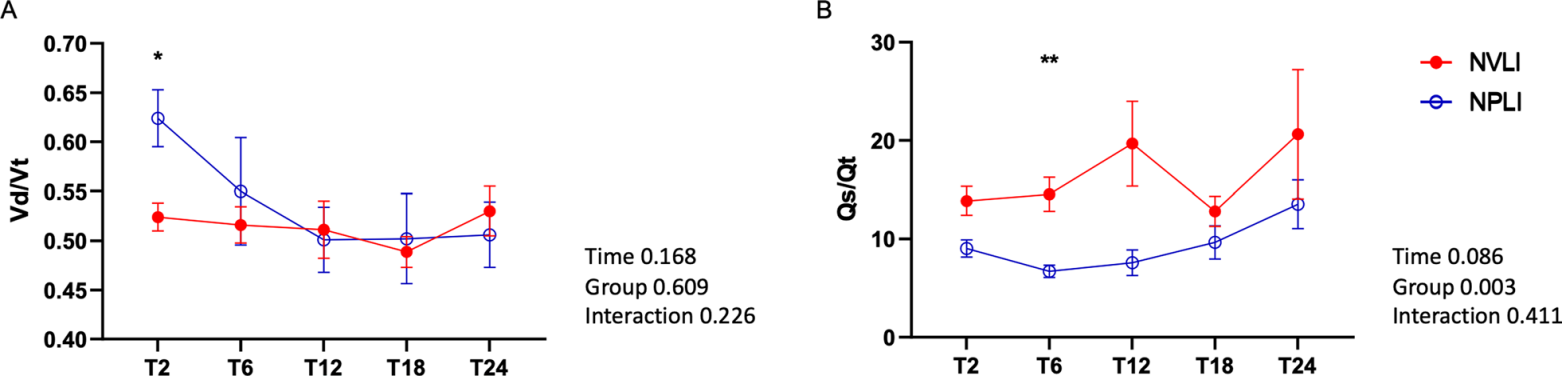
Classical gas exchange-based measures of V/Q mismatch. Dead space (**A**) measured by volumetric capnography and shunt (**B**) calculated by Berggren equation. Data are expressed as mean ± standard error. For each dependent variable, mixed-effect model for repeated measures was performed to detect significant differences between groups over time (Sidak’s post hoc test). P-values are reported in the graphs. *p < 0.05, **p < 0.01. Vd/Vt, physiological dead space; Qs/Qt, intrapulmonary shunt; NVLI, non-ventilated lung injury; NPLI, non-perfused lung injury

Physiological dead space measured by volumetric capnography was significantly higher in the NPLI group only at T2, then the values became identical for the two study groups (Fig. 7A); the shunt fraction calculated with the Berggren equation was very low in both groups, and only slightly higher in the NVLI group, as compared to NPLI group (Fig. 7B). This suggests that compensatory mechanisms, such as HPV and hypocapnic bronchoconstriction, may have dynamically compensated for gas exchange-based measures of V/Q mismatch, impairing their reliability.

#### Pulmonary vascular dysfunction

Pulmonary artery pressures and resistances were higher and the pulmonary artery compliance low in the NVLI group, compared to NPLI group (Fig. 8.1A–E). Interestingly, regional pulmonary vascular resistance (PVR) was high and pulmonary artery compliance low in the left lung of the NVLI group (Fig. 8.2A, B), compared to perfused regions of the NVLI and NPLI group. Together, these results suggest higher risk of vascular shear stress in the left lung of the NVLI group. Lack of pulmonary blood flow in the left lung of the NPLI group, instead, potentially prevented local vascular shear stress.

**Fig. 8:**
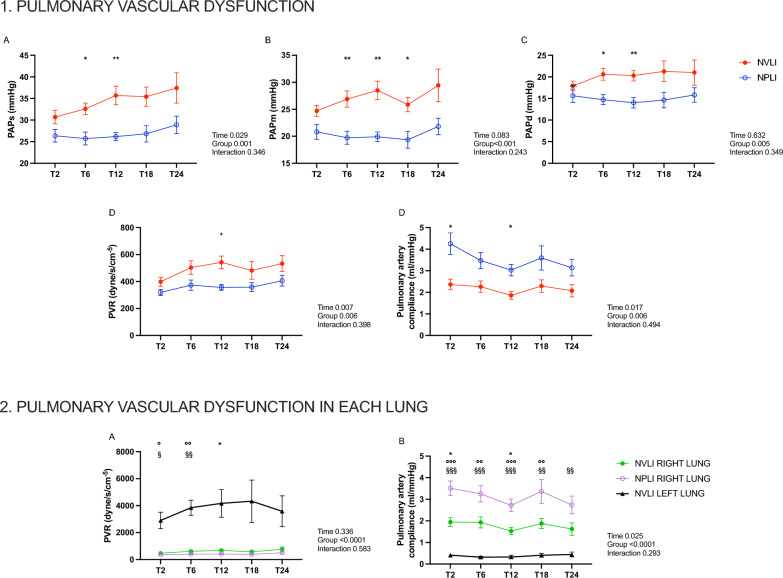
Pulmonary vascular dysfunction. Panel 1: systolic (**A**), mean (**B**) and diastolic (**C**) arterial pulmonary pressure, pulmonary vascular resistances (**D**) and pulmonary artery compliance (**E**) along the experiment in each group. Panel 2: pulmonary vascular resistance (**A**) and pulmonary artery compliance (**B**) in each lung from the two study groups. Left lung pulmonary vascular resistances and pulmonary artery compliance were not represented in the NPLI group because the left pulmonary artery was excluded from perfusion. Data are expressed as mean ± standard error. For each dependent variable, mixed-effect model for repeated measures was performed to detect significant differences between groups over time (Sidak’s post hoc test). *P*-values are reported in the graphs. Panel 1: *p < 0.05, **p < 0.01 Panel 2: *p < 0.05 NVLI RIGHT vs NPLI RIGHT, °*p* < 0.05, °°*p* < 0.01 and °°°*p* < 0.001 NVLI RIGHT VS NVLI LEFT, ^§^*p* < 0.05, ^§§^*p* < 0.01 and ^§§§^*p* < 0.001 NPLI RIGHT VS NVLI LEFT. NVLI, non-ventilated lung injury; NPLI, non-perfused lung injury; PAPs, systolic pulmonary artery pressure; PAPm, mean pulmonary artery pressure; PAPd, diastolic pulmonary artery pressure; PVR, pulmonary vascular resistances

**Fig. 9:**
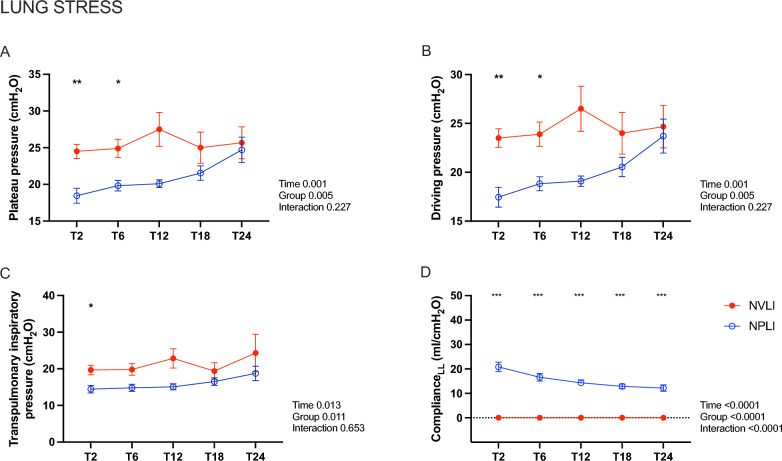
Static and dynamic inspiratory lung stress. Plateau pressure (**A**) driving pressure (**B**) and transpulmonary inspiratory pressure (**C**) as measures of stress applied to the respiratory system (**A** and **B**) and the lung (**C**), respectively. Compliance for the left lung in each group (D) as a measure of regional stress. Data are expressed as mean ± standard error. For each dependent variable, mixed-effect model for repeated measures was performed to detect significant differences between groups over time (Sidak’s post hoc test). *P*-values are reported in the graphs. *p < 0.05, **p < 0.01, ****p < 0.001. LL, left lung; NVLI, non-ventilated lung injury; NPLI, non-perfused lung injury

*Inspiratory lung stress.* Values of lung stress, potentially inducing barotrauma in the ventilated left lung of the NPLI group, worsened along the experiment (Fig. 9A–C), specifically due to a decrease of compliance of the left lung (Fig. 9D). Left lung compliance could have worsened following local hypocapnic bronchoconstriction and/or development of regional lung injury. Absence of ventilation to the left lung, instead, prevented risk of direct regional barotrauma in the NVLI group. Additional physiological data along the experiments can be found in Supplementary Table 1. Physiological data for the Control group are reported in Supplementary Table 2.

### Proteomic profile of lung injury

**Fig. 10:**
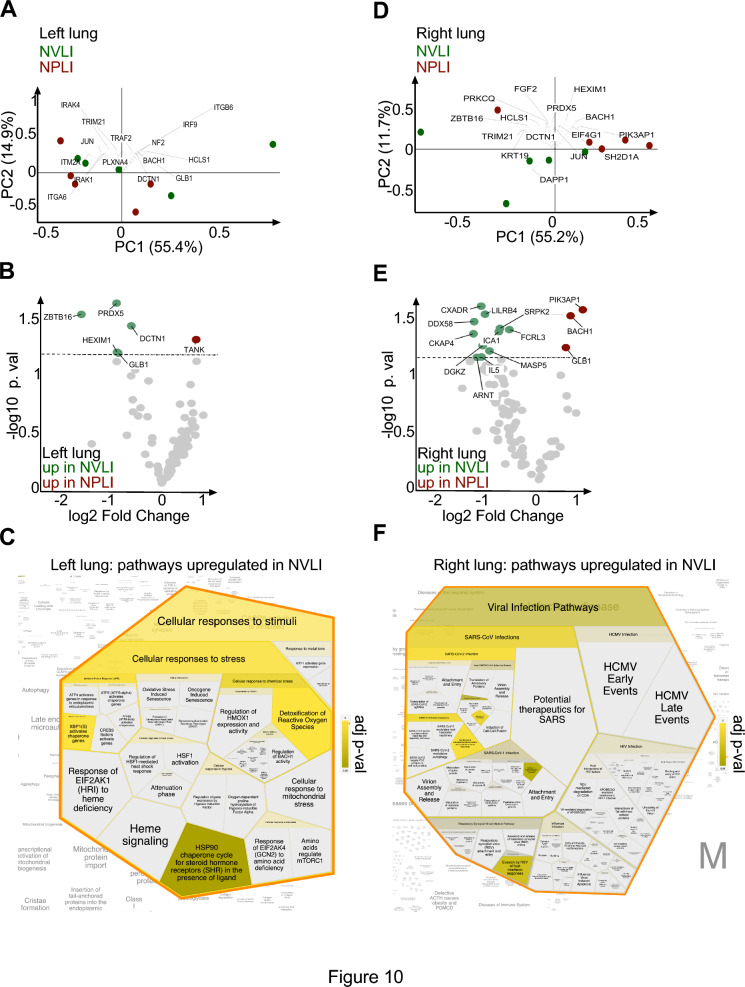
Proteomic analysis of left and right lung tissue from NVLI and NPLI groups. Analysis of immune response proteins was performed with the Olink platform in left (**A**–**C**) and right (**D**–**F**) lung. **A**, **D** Unsupervised principal component (PC) analysis; the proteins which account for the most variance within the indicated dataset (loadings) are shown. **B**, **E** Volcano plot was used to show differentially expressed proteins (*p* < 0.05; log2 fold change >|0.5|) between NVLI and NPLI in left (**B**) or right (**E**) lung. Overrepresented pathway analysis was performed with Reactome for proteins upregulated in NVLI left (**C**) or right (**F**) lung. See also Figure Online 1 for complete pathways results. NVLI, non-ventilated lung injury; NPLI, non-perfused lung injury

Globally, unsupervised analysis of the left lungs did not show major changes at the protein level able to discriminate cases of the NVLI from the NPLI groups (Fig. 10), suggesting that the two lung injuries share some immune response signaling. Nevertheless, we found significantly different proteins upregulated in the NVLI left lung compared with NPLI (Fig. 10), such as the p*eroxiredoxin-5* (PRDX5, an antioxidant protein involved in inflammation), *zinc finger and BTB domain-containing protein 16* (ZBTB16, a promoter of inflammatory pulmonary fibrosis), *Dynactin subunit 1* (DCTN1, essential for intracellular transport), *HEXIM1* (a negative regulator of the transcription elongation factor b) and *Beta-galactosidase* (GLB1, an enzyme located within lysosomes that breaks keratan sulfate, which has protective effects in the small airway). PRDX5 and DCTN1 were also upregulated in the left lung compared with its right counterpart within the NVLI group (Online Fig. S3A). Pathways analysis showed that these proteins are involved in cellular responses to stress (Fig. 10 and Online Fig. S2).

On the other hand, only the *TRAF family member-associated NF-kappa-B activator* (TANK), a factor involved in the Toll-like receptor signaling [13], was upregulated in the left lungs of the NPLI group (Fig. 10).

### Right lung injury

**Fig. 11:**
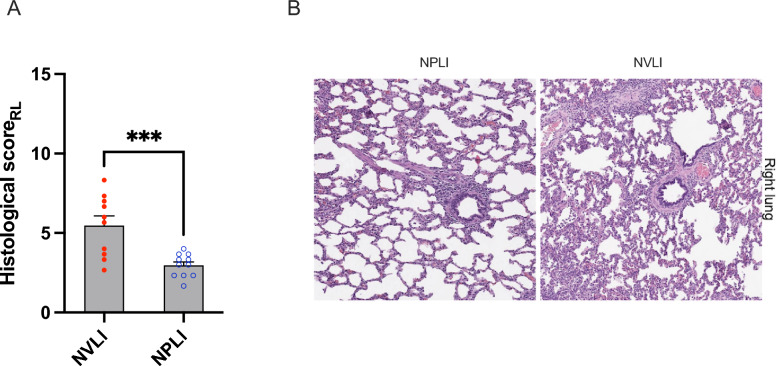
Right lung injury histological score. Right lung histological score (**A**) showed significantly higher values in NVLI vs. NPLI group. Representative microphotographs (H&E, scale bar: 300 μm) for the right lung (**B**) of the NVLI group (right panel) and for the NPLI group (left panel) are shown. Data are expressed as scatter plot with bars and error bars (mean ± SEM). Statistical analysis is performed by unpaired *t*-test or Mann–Whitney *U* test, as appropriated, p-values are reported in the graphs. ***p < 0.001, ***p < 0.0001; RL, right lung; NVLI, non-ventilated lung injury; NPLI, non-perfused lung injury

Animals from the NVLI group developed histological evidence of injury to the right lung, too, albeit with lower severity than the left one (Fig. 11). The right lung of NPLI, instead, showed low values of histological injury, like normal uninjured lungs. The score sub-items characterizing the right lung of the NVLI group (alveolar hemorrhage, alveolar macrophage proliferation, hyaline membrane formation) (Figure Online 4) are well-known histological features of classical VILI. Alveolar pro-inflammatory cytokines levels confirmed more intense injury in the right lung of NVLI vs. NPLI (Figure Online 5). See Online Supplements for details on physiologic, imaging and proteomic profile of right lung injury.

## Discussion

This study showed that NVLI and NPLI are different, both for the target and the distal lung regions, in terms of physiologic derangements causing lung injury (i.e., V/Q mismatch, pulmonary vascular dysfunction, lung stress, inflammation); in the severity of lung injury; and in alveolar- and tissue-specific proteomic expression profile. NVLI presents larger and specific pathophysiological derangements, leading to more severe bilateral lung injury and more intense inflammatory reaction. Explorative proteomic analysis within the target left lung discovered specific proteins associated with NVLI, with protective and injurious functions, thus opening novel research avenues for attempting prevention and/or treatment.

Regarding damage to non-ventilated collapsed lung areas, some authors suggested that such zones could be spared from VILI [3, 14, 15], but these areas might be susceptible to damage caused by alveolar collapse and hypoperfusion, as shown in our study. In the NVLI group, we demonstrated inflammation activation in the non-ventilated lung, evidenced by neutrophil infiltration, macrophage proliferation and elevated levels of cytokines (IL-1β and IL-6 in BAL). Previous studies showed that the combination of low alveolar oxygen tension and reduced perfusion leads to ischemic insult and inflammation in atelectatic regions [16, 17]. These areas develop endothelial [18] and epithelial damage, with microvascular alterations and dysfunction of type I and II pneumocytes, leading to alveolar edema [17]. In our NVLI model, histological analysis confirmed interstitial congestion and alveolar hemorrhage in non-ventilated units. Moreover, contralateral flow redistribution from the non-ventilated to ventilated lung due to hypoxic vasoconstriction resulted in dramatic vascular dysfunction and perfusion reduction, potentially exacerbating damage [16, 19, 20]. Of note, the proteomic analysis in our model of collapsed lung showed increased expression of anti-proliferative and protective molecular pathways, signifying that a defensive response to the damage coexists with the harmful inflammatory process.

Mechanisms triggering damage in non-perfused lung differ from the ones of non-ventilated injury. Experimental findings suggest that alveolar hypocapnia resulting from ventilation of non-perfused units might trigger apoptosis of type II pneumocytes and surfactant dysfunction, leading to a localized decrease in compliance [24] and bronchoconstriction, sudden compliance reduction, and possible increase in ventilation of perfused areas [12, 22]. The same was verified in our NPLI group, which developed a progressive deterioration of compliance in the left non-perfused lung with redistribution of ventilation to the right perfused lung. Correction of alveolar hypocapnia by CO_2_ supplementation [5, 21, 23–28] counteracts these changes and prevents tissue damage [5, 21, 25–27], and worsening of compliance [5, 18, 23]. The combination of hypocapnia and impaired alveolar oxygenation which result from extensive derangements of V/Q matching likely explain the more severe lung injury in the left lung of NVLI group.

Inflammatory events also differed between NVLI and NPLI: halted perfusion might have prevented delivery and infiltration of inflammatory cells, which were lower in the left lung of the NPLI group. More intense interstitial congestion, and alveolar hemorrhage characterizing NPLI, instead, validate previous studies [24, 25]. The high expression of TANK in left lung tissue, a protein with anti-inflammatory properties, may indicate concomitant restorative mechanisms acting to mitigate lung injury in the NPLI.

In our study, NVLI group developed lung damage on both sides, while the right lung of NPLI was spared from injury. In the NVLI group, the right lung receives 100% of ventilation and approximately 80% of blood flow and is relatively more overstretched than overperfused (ventilation predominantly distributed to areas of high V/Q), with higher lung stress and lower compliance leading to higher risk of VILI [10, 29], in comparison to NPLI. The high fraction of wasted ventilation in the right side of the NVLI group suggested that regional alveolar hypocapnia, coupled with elevated ventilation pressures and overperfusion [30], may contribute additively to the development of damage [21, 23, 25, 28]. Mechanistically, the injury in NVLI group induced upregulation of proteins involved in cellular response to stress, in the left lung, and to a general inflammatory state, in the right lung.

Our study presents a significant strength: it reproduces an in vivo model of shunt and dead space, with non-ventilated and non-perfused regions clearly compartmentalized. This allows for a more detailed characterization of pathophysiological alterations using advanced monitoring systems, as well as a precise assessment of the injury affecting different lung regions, supporting the formulation of hypotheses regarding the underlying mechanisms.

Our injury models, defined by altered ventilation–perfusion (V/Q) ratios, provide a reproducible and controlled experimental simplification of clinical scenarios involving non-ventilated or non-perfused lung regions. Many pulmonary conditions—such as pneumonia, absorption atelectasis, pulmonary contusion, pneumothorax, or one-lung ventilation during thoracic surgery—are characterized by areas excluded from ventilation, leading to collapse and redistribution of ventilation to adjacent regions. On the other hand, perfusion defects are typically observed in pulmonary embolism and, notably, in ARDS, where vascular microthrombi contribute to perfusion heterogeneity. ARDS represents a particularly complex syndrome due to its marked spatial heterogeneity in both ventilation and perfusion, with coexisting collapsed, non-ventilated, and non-perfused areas. In this context, V/Q mismatch is not only a hallmark of disease severity [31], but also a prognostic factor, with several studies linking the extent of shunt and dead space to increased mortality [2, 32].

Our experiment has some limitations: 1. we used relatively high tidal volume mechanical ventilation with low PEEP to reduce time to lung injury. Moreover, according to our previous study [4], the choice of high tidal volume was also intended to limit hypercapnia in the NVLI group (which may lead to beneficial effects). We realize that use of lung protective ventilation could have led to different intensity of injury and/or longer time needed to develop the same degree of injury. 2. We hypothesized two important mechanisms of damage (tissue hypoxia and alveolar hypocapnia) based on V/Q mismatch but couldn’t directly measure them. 3. We opted to exclude the left lung from ventilation or perfusion; whether excluding the right lung would have resulted in different outcomes remains unknown. 4. The proteomic analysis is explorative but opens novel interesting research avenues to validate findings. 5. We used only female pigs as in previous studies, as the effect of gender-based differences was beyond the scope of this study.

## Conclusions

The presence of extensive non-ventilated or non-perfused lung regions induces local and distal difference in the physiological responses, severity of injury, inflammatory reaction and proteomic profile. NVLI, characterized by collapse, lung hypoxia and hypoperfusion in the non-ventilated side, and stress, overperfusion and high dead space ventilation in the contralateral lung seems more severe than NPLI, in which hypoperfusion and alveolar hypocapnia injure the target lung, while the contralateral remains spared. Proteomic analysis suggested initial activation of protective pathways in both groups, that might be, eventually, re-enforced to halt injury.

## Supplementary Information

Additional file 1. Supplementary Table 1. Physiological variables along the experiment in the NVLI and NPLI groups. Supplementary Table 2. Histological and physiological variables at the end of experiment in the Control group (*n* = 6). Figure Online 1. Representative images of the lungs at the end of the experiment. NVLI: Non-Ventilated Lung Injury; NPLI: Non-Perfused Lung Injury. Figure Online 2. Pathway analysis was performed in Reactome using the overrepresentation function for proteins upregulated in NVLI left and right lung compared with NPLI. NVLI: Non-Ventilated Lung Injury; NPLI: Non-Perfused Lung Injury. Figure Online 3. The Immune Response proteome in NVLI and NPLI groups. The supervised heatmaps show protein expression in right and left lung samples. Significantly different proteins in the right (RL) and left (LL) lung from NVLI (**A**) and NPLI (**B**) groups are reported in the tables along with the mean expression value of and p-value. NVLI: Non-Ventilated Lung Injury; NPLI: Non-Perfused Lung Injury. Figure Online 4. Right lung histological injury score sub-items. The right lung of the NVLI group showed more alveolar hemorrhage (**A**), alveolar macrophage proliferation (**B**) and hyaline membrane formation (**C**) compared to the right lung of the NPLI group. Data are expressed as scatter plot with bars and error bars (mean ± SEM). Statistical analysis is performed by unpaired t-test or Mann–Whitney U test, as appropriated, p-values are reported in the graphs. *p < 0.05, ***p < 0.001, ****p < 0.0001. RL, right lung; NVLI, non-ventilated lung injury; NPLI, non-perfused lung injury. Figure Online 5. Cytokines concentration from the right BAL fluid. Alveolar concentration of IL-1β and IL-6 was measured in the right lung (**A** and **B** respectively) of each study group. Data are expressed as scatter plot with bars (mean ± SEM) where each sample is a dot. Statistical analysis is performed by unpaired *t*-test or Mann–Whitney *U* test, as appropriated. *p < 0.05, ***p < 0.001, ****p < 0.0001. NVLI, non-ventilated lung injury; NPLI, non-perfused lung injury. Figure Online 6. EIT analysis of distribution of ventilation and perfusion to the different V′/Q compartments in the right lung. **1** EIT-measured fraction of ventilation reaching the dead space (**A**), high V′/Q (**B**) normal V′/Q (**C**) and low V′/Q (**D**) units in the right lung. In the NVLI group the ventilation reached mainly dead space and high V’/Q compartments, while in the NPLI group the ventilation of the normal V’/Q compartment was higher. Panel 2: EIT-measured fraction of perfusion reaching the high V′/Q (**A**), normal V′/Q (**B**), low V′/Q (**C**) and shunt (**D**) units in the right lung. The right lung of the NVLI group was highly unmatched with around 80% of perfusion distributing to high V′/Q compartment, while in the NPLI group the perfusion reached more homogeneously high and normal V′/Q compartments. Data are expressed as mean ± SEM. Comparisons are obtained with mixed-effect model for repeated measures followed by Sidak’s post hoc test with groups and time as independent factors and group × time interaction. *P*-values are reported in the graphs. NVLI, non-ventilated lung injury; NPLI, non-perfused lung injury; V: ventilation; P: perfusion; V/Q ventilation to perfusion.

## Data Availability

The datasets used during the current study are available from the corresponding author on reasonable request.

## Abbreviations

ARNT: Aryl hydrocarbon receptor nuclear translocator
BAL: Broncho-alveolar lavage
CXADR: Coxsackievirus and adenovirus receptor
DGKZ: Diacylglycerol kinase Z
DCTN1: Dynactin subunit 1
DDX58: Antiviral innate immune response receptor RIG-I
EIT: Electrical impedance tomography
FCRL3: Fc receptor-like protein 3
FiO_2_: Inspired O_2_ fraction
GLB1: Beta-galactosidase
ICA1: Islet cell autoantigen 1
I:E: Inspiration to expiration ratio
IL-1β: Interleukin 1β
IL-6: Interleukin 6
LLRB4: Leukocyte immunoglobulin-like receptor subfamily B member 4
NPLI: Non-perfused lung injury
NVLI: Non-ventilated lung injury
PCA: Primary component analysis
PEEP: Positive end-expiratory pressure
PIK3AP1: Phosphoinositide 3-kinase adapter protein 1
PRDX5: Peroxiredoxin-5
TANK: TRAF family member-associated NF-kappa-B activator
VILI: Ventilator-induced lung injury
V/Q: Ventilation/perfusion
ZBTB16: Zinc finger and BTB domain-containing protein 16

## References

[1] Slobod D, Damia A, Leali M, Spinelli E, Mauri T (2022) Pathophysiology and clinical meaning of ventilation-perfusion mismatch in the acute respiratory distress syndrome. Biology (Basel) 12(1):67. 10.3390/biology12010067 36671759 10.3390/biology12010067 PMC9855693

[2] Nuckton TJ, Alonso JA, Kallet RH et al (2002) Pulmonary dead-space fraction as a risk factor for death in the acute respiratory distress syndrome. N Engl J Med 346(17):1281–1286. 10.1056/NEJMoa012835 11973365 10.1056/NEJMoa012835

[3] Tsuchida S, Engelberts D, Peltekova V et al (2006) Atelectasis causes alveolar injury in nonatelectatic lung regions. Am J Respir Crit Care Med 174(3):279–289. 10.1164/rccm.200506-1006OC 16675780 10.1164/rccm.200506-1006OC

[4] Spinelli E, Damia A, Damarco F et al (2024) Pathophysiological profile of non-ventilated lung injury in healthy female pigs undergoing mechanical ventilation. Commun Med (Lond). 4(1):18. 10.1038/s43856-024-00449-3 38361130 10.1038/s43856-024-00449-3 PMC10869686

[5] Marongiu I, Spinelli E, Scotti E et al (2021) Addition of 5% CO_2_ to inspiratory gas prevents lung injury in an experimental model of pulmonary artery ligation. Am J Respir Crit Care Med 204(8):933–942. 10.1164/rccm.202101-0122OC 34252009 10.1164/rccm.202101-0122OC PMC8534619

[6] Cressoni M, Caironi P, Polli F et al (2008) Anatomical and functional intrapulmonary shunt in acute respiratory distress syndrome. Crit Care Med 36(3):669–675. 10.1097/01.CCM.0000300276.12074.E1 18091555 10.1097/01.CCM.0000300276.12074.E1

[7] Spinelli E, Perez J, Chiavieri V et al (2024) Pathophysiological markers of acute respiratory distress syndrome severity are correlated with ventilation-perfusion mismatch measured by electrical impedance tomography. Crit Care Med. 10.1097/CCM.0000000000006458 39445936 10.1097/CCM.0000000000006458

[8] Bruinooge AJG, Mao R, Gottschalk TH et al (2022) Identifying biomarkers of ventilator induced lung injury during one-lung ventilation surgery: a scoping review. J Thorac Dis 14(11):4506–4520. 10.21037/jtd-20-2301 36524064 10.21037/jtd-20-2301 PMC9745541

[9] Pascut D, Giraudi PJ, Banfi C et al (2023) Proteome profiling identifies circulating biomarkers associated with hepatic steatosis in subjects with Prader-Willi syndrome. Front Endocrinol (Lausanne). 14:1254778. 10.3389/fendo.2023.1254778 38034016 10.3389/fendo.2023.1254778 PMC10684934

[10] Protti A, Cressoni M, Santini A et al (2011) Lung stress and strain during mechanical ventilation: any safe threshold? [published correction appears in Am J Respir Crit Care Med. 2012 Jan 1;185(1):115]. Am J Respir Crit Care Med 183(10):1354–1362. 10.1164/rccm.201010-1757OC 21297069 10.1164/rccm.201010-1757OC

[11] Spinelli E, Pesenti A, Lopez G et al (2022) Inhaled CO2 vs. hypercapnia obtained by low tidal volume or instrumental dead space in unilateral pulmonary artery ligation: any difference for lung protection. Front Med (Lausanne). 9:901809. 10.3389/fmed.2022.901809 35669918 10.3389/fmed.2022.901809 PMC9163369

[12] Langer T, Castagna V, Brusatori S et al (2019) Short-term physiologic consequences of regional pulmonary vascular occlusion in pigs. Anesthesiology 131(2):336–343. 10.1097/ALN.0000000000002735 31094756 10.1097/ALN.0000000000002735

[13] Clark K, Takeuchi O, Akira S, Cohen P (2011) The TRAF-associated protein TANK facilitates cross-talk within the IkappaB kinase family during Toll-like receptor signaling. Proc Natl Acad Sci USA 108(41):17093–17098. 10.1073/pnas.1114194108 21949249 10.1073/pnas.1114194108 PMC3193242

[14] Chu EK, Whitehead T, Slutsky AS (2004) Effects of cyclic opening and closing at low- and high-volume ventilation on bronchoalveolar lavage cytokines. Crit Care Med 32(1):168–174. 10.1097/01.CCM.0000104203.20830.AE 14707576 10.1097/01.CCM.0000104203.20830.AE

[15] Wakabayashi K, Wilson MR, Tatham KC, O’Dea KP, Takata M (2014) Volutrauma, but not atelectrauma, induces systemic cytokine production by lung-marginated monocytes. Crit Care Med 42(1):49–57. 10.1097/CCM.0b013e31829a822a 10.1097/CCM.0b013e31829a822a 23963135

[16] Tojo K, Nagamine Y, Yazawa T et al (2015) Atelectasis causes alveolar hypoxia-induced inflammation during uneven mechanical ventilation in rats. Intensive Care Med Exp 3(1):1–17. 10.1186/s40635-015-0056-z 26215820 10.1186/s40635-015-0056-z PMC4480346

[17] Duggan M, Kavanagh BP (2005) Pulmonary atelectasis: a pathogenic perioperative entity. Anesthesiology 102(4):838–854. 10.1097/00000542-200504000-00021 15791115 10.1097/00000542-200504000-00021

[18] Duggan M, McCaul CL, McNamara PJ, Engelberts D, Ackerley C, Kavanagh BP (2003) Atelectasis causes vascular leak and lethal right ventricular failure in uninjured rat lungs. Am J Respir Crit Care Med 167(12):1633–1640. 10.1164/rccm.200210-1215OC 12663325 10.1164/rccm.200210-1215OC

[19] Lohser J, Slinger P (2015) Lung injury after one-lung ventilation: a review of the pathophysiologic mechanisms affecting the ventilated and the collapsed lung. Anesth Analg 121(2):302–318. 10.1213/ANE.0000000000000808 26197368 10.1213/ANE.0000000000000808

[20] Sivrikoz MC, Tunçözgür B, Çekmen M et al (2002) The role of tissue reperfusion in the reexpansion injury of the lungs. Eur J Cardio Thorac Surg 22(5):721–727. 10.1016/S1010-7940(02)00447-5 10.1016/s1010-7940(02)00447-5 12414037

[21] Kiefmann M, Tank S, Tritt MO et al (2018) Dead space ventilation promotes alveolar hypocapnia reducing surfactant secretion by altering mitochondrial function. Thorax. 10.1136/thoraxjnl-2018-211864 10.1136/thoraxjnl-2018-211864 30636196

[22] Tsang JYC, Lamm WJE, Swenson ER, Tsang JYC (2009) Regional CO2 tension quantitatively mediates homeostatic redistribution of ventilation following acute pulmonary thromboembolism in pigs. J Appl Physiol 107:755–762. 10.1152/japplphysiol.00245.2009.-Previous 19608933 10.1152/japplphysiol.00245.2009

[23] Ingram RHJ (1985) Effects of airway versus arterial CO2 changes on lung mechanics in dogs. J Appl Physiol 38(4):603–607. 10.1152/jappl.1975.38.4.603 10.1152/jappl.1975.38.4.603 1141090

[24] Edmunds H, Holm JC (1969) Effect of inhaled CO2 on hemorrhagic consolidation due to unilateral pulmonary arterial ligation. J Appl Physiol 26(6):710–715 5786399 10.1152/jappl.1969.26.6.710

[25] Kolobow T, Spragg RG, Pierce JE (1981) Massive pulmonary infarction during total cardiopulmonary bypass in unanesthetized spontaneously breathing lambs. Int J Artif J 4(2):76–81 6792084

[26] Shepard JW, Hauer D, Miyai K, Moser KM (1980) Lamellar body depletion in dogs undergoing pulmonary artery occlusion. J Clin Invest 66(1):36–42. 10.1172/JCI109832 6772668 10.1172/JCI109832 PMC371502

[27] Bayindir O, Akpinar B, Özbek U et al (2000) The hazardous effects of alveolar hypocapnia on lung mechanics during weaning from cardiopulmonary bypass. Perfusion 15(1):27–31. 10.1177/026765910001500105 10676865 10.1177/026765910001500105

[28] Laffey JG, Engelberts D, Kavanagh BP (2000) Injurious effects of hypocapnic alkalosis in the isolated lung. Am J Respir Crit Care Med 162:399–405 10934060 10.1164/ajrccm.162.2.9911026

[29] Slutsky AS (1999) Lung injury caused by mechanical ventilation. Chest 116(1):9S-15S 10424561 10.1378/chest.116.suppl_1.9s-a

[30] Hotchkiss JR, Blanch L, Naveira A et al (2001) Relative roles of vascular and airspace pressures in ventilator-induced lung injury. Crit Care Med 29(8):1593–1598. 10.1097/00003246-200108000-00016 11505134 10.1097/00003246-200108000-00016

[31] Spinelli E, Kircher M, Stender B et al (2021) Unmatched ventilation and perfusion measured by electrical impedance tomography predicts the outcome of ARDS. Crit Care 25(1):192. 10.1186/s13054-021-03615-4 34082795 10.1186/s13054-021-03615-4 PMC8173510

[32] Bellani G, Laffey JG, Pham T et al (2016) Epidemiology, patterns of care, and mortality for patients with acute respiratory distress syndrome in intensive care units in 50 countries [published correction appears in JAMA. 2016 Jul 19;316(3):350]. JAMA 315(8):788–800. 10.1001/jama.2016.0291 26903337 10.1001/jama.2016.0291

