# Supplementary material for: Physiology, imaging and proteomics of non-ventilated vs. non-perfused lung injury: an experimental study
Source: Intensive Care Med Exp. 2025 Jun 23;13:63. doi: 10.1186/s40635-025-00772-6 (PMC12185848; doi:10.1186/s40635-025-00772-6)
Supplement: Supplementary file 1 — Additional file 1. Supplementary Table 1. Physiological variables along the experiment in the NVLI and NPLI groups. Supplementary Table 2. Histological and physiological variables at the end of experiment in the Control group (n = 6). Figure Online 1. Representative images of the lungs at the end of the experiment. NVLI: Non-Ventilated Lung Injury; NPLI: Non-Perfused Lung Injury. Figure Online 2. Pathway analysis was performed in Reactome using the overrepresentation function for proteins upregulated in NVLI left and right lung compared with NPLI. NVLI: Non-Ventilated Lung Injury; NPLI: Non-Perfused Lung Injury. Figure Online 3. The Immune Response proteome in NVLI and NPLI groups. The supervised heatmaps show protein expression in right and left lung samples. Significantly different proteins in the right (RL) and left (LL) lung from NVLI (A) and NPLI (B) groups are reported in the tables along with the mean expression value of and p-value. NVLI: Non-Ventilated Lung Injury; NPLI: Non-Perfused Lung Injury. Figure Online 4. Right lung histological injury score sub-items. The right lung of the NVLI group showed more alveolar hemorrhage (A), alveolar macrophage proliferation (B) and hyaline membrane formation (C) compared to the right lung of the NPLI group. Data are expressed as scatter plot with bars and error bars (mean ± SEM). Statistical analysis is performed by unpaired t-test or Mann–Whitney U test, as appropriated, p-values are reported in the graphs. *p < 0.05, ***p < 0.001, ****p < 0.0001. RL, right lung; NVLI, non-ventilated lung injury; NPLI, non-perfused lung injury. Figure Online 5. Cytokines concentration from the right BAL fluid. Alveolar concentration of IL-1β and IL-6 was measured in the right lung (A and B respectively) of each study group. Data are expressed as scatter plot with bars (mean ± SEM) where each sample is a dot. Statistical analysis is performed by unpaired t-test or Mann–Whitney U test, as appropriated. *p < 0.05, ***p < 0.001, ****p < 0 [file 40635_2025_772_MOESM1_ESM.docx]

**Physiology, imaging and proteomics of non-ventilated vs. non-perfused lung injury: an experimental study**

Anna Damia et al.

**Online Supplement**

**Supplementary methods**

*Animal preparation*. In compliance with local recommendations, pigs arrived at the experimental facility the day before the start of the study and fasted overnight with free access to water. Sedation was administered by intramuscular injection of medetomidine 0.025 mg/kg and tiletamine/zolazepam 5 mg/kg. Then, an auricular vein was cannulated and, after administration of cefazoline 1 g and tramadol 50 mg, continuous intravenous (IV) infusion of propofol was titrated to maintain the animal on spontaneous breathing and SpO2 100% while on additional oxygen via face mask. Surgical tracheostomy was performed in the supine position under additional local anesthesia (lidocaine 2%). After a left sided double-lumen endobronchial tube of 37 Fr (for the NVLI group) or an endotracheal tube of 7.5 mm (for the NPLI) was inserted through the tracheostomy and fixed, mechanical ventilation (Evita Infinity V500, Dräger, Lübeck, Germany) was started and general anesthesia and neuromuscular blockade were maintained by IV propofol 5-10 mg/kg/h, medetomidine 2.5-10.0 μg/kg/h and pancuronium bromide 0.3-0.5 mg/kg/h. Correct positioning of the endobronchial tube was assessed with a single-use fiberscope (Ambu aScope™ 4 Broncho Slim, Ambu, Netherlands). During the whole experiment, oxygen saturation, heart rate, invasive blood pressure, central venous pressure, pulmonary artery pressure and ventilatory waveforms were monitored. Depth of anesthesia was adjusted to ensure no sign of distress, such as unexplained tachycardia, arterial hypertension and/or horripilation. External warming was provided when core body temperature fell below 37.5°C, as well as external cooling when core body temperature rose above 40°C.

Ringer lactate was administered at 100 ml/h during catheters positioning and reduced to 50 ml/h along the whole study, aiming at a zero fluid balance (cumulative fluid balance was recorded).

Cefazoline 1 g IV and tramadol 50 mg IV were administered every 12 hours. Low molecular weight heparin 2000 IU was administered subcutaneously once per day.

*Instrumentation*. Vascular accesses were obtained by surgical exposure. An arterial catheter (Arrow® Seldinger, 18 G 8 cm, Prodimed, Teleflex, Ireland) was inserted in the left common carotid artery. A three-lumen central venous catheter (Arrow®, 7 Fr, Teleflex, Ireland) and a pulmonary artery catheter (Swan Ganz®, 5 Fr, Edwards, USA) were introduced in the left external jugular vein. Positioning of pulmonary artery catheter was guided by visualization of pulmonary artery and wedge pressures. A multifunction nasogastric catheter with esophageal balloon (Nutrivent^TM^, Sidam, Italy) was inserted and inflated with the recommended volume of air. Correct positioning and calibration were confirmed by the standard occlusion test with external compressions.

*Exclusion of the left lung from ventilation (NVLI group) or from perfusion (NPLI group).* At the end of instrumentation, the correct positioning of the endobronchial tube was assessed again using the bronchoscope in animals of the NVLI group and the left lung was excluded from ventilation. In animals of the NPLI group, instead, surgical ligation of the left pulmonary artery was performed as follows: a 10 cm left thoracotomy was done at the level of the 4th or 5th intercostal space; the main left pulmonary artery was isolated and progressively (5 minutes) occluded and then ligated with a non-absorbable silk suture thread. A chest tube used to evacuate residual air from the pleural cavity and removed during a recruitment maneuver at the time of chest closure.

*Study measurements*. Pulsoxymetry, heart rate, invasive arterial pressure and pulmonary artery pressure were continuously monitored. End-tidal CO_2_ and physiological dead space were measured through volumetric capnography connected to the ventilator circuit. A 16 electrodes EIT belt was positioned around the swine's chest, connected to an EIT monitor (PulmoVista® 500, Dräger, Lübeck, Germany) and left in place throughout the study period.

*EIT analysis.* EIT data were recorded at 50 Hz and stored for offline analysis. EIT ventilation and perfusion maps were obtained by dedicated software. We split the EIT images into two same size regions of interests on the horizontal axis, roughly corresponding to the right and left lungs. For ventilation maps, we averaged values over 5 consecutive respiratory cycles. For each lung, the regional compliance was calculated as the ratio between the % of tidal volume to the lung (left or right) divided by the transpulmonary driving pressure.

EIT perfusion maps were derived from offline analysis of the time-impedance curve obtained by first pass of a 10 ml-bolus of 5% saline solution injected during an end-inspiratory occlusion, as previously described [1]. Perfusion of the non-ventilated left lung was quantified as the fraction of blood flow perfusing the non-ventilated pixels in the left hemithorax.

For calculating ventilation/perfusion matching, in the ventilation and perfusion maps we considered as non-ventilated and non-perfused, respectively, those pixels whose values laid below the 20% of the maximum value within the map. This is consistent with previous work [2, 3]. Each map was then normalized to express percent ventilation and perfusion, which were then multiplied by measured minute volume and cardiac output, respectively, to obtain absolute estimates in ml/min. Element-wise division was then performed to obtain a third map, reflecting regional ventilation to perfusion (V’/Q) ratio. Thresholds were applied to the map and five compartments were defined, as previously described [4]. In brief:

- pixels whose V’/Q values exceeded 10 were considered as dead space;
- pixels in the 1.25÷10 range as having high V’/Q;
- pixels in the 0.8÷1.25 range as normal;
- pixels in the 0.1÷0.8 range as having low V’/Q;
- pixels below 0.1 as shunt;

Ventilation and perfusion to each V’/Q compartment was then calculated as the sum of fractions of corresponding pixels in the ventilation and perfusion maps, respectively.

*Pulmonary hemodynamics:* At each timepoint we recorded systolic, diastolic and mean pulmonary artery pressures (PAPS, PAPD, PAPM); wedge pressure (WP) at end expiration; cardiac output (CO) via thermodilution technique (Vigilance, Baxter Edwards Critical Care, Edwards E6 Lifesciences, USA); heart rate (HR); central venous pressure (CVP) at end expiration. In order to assess the status of the pulmonary circulation of each lung we calculated regional pulmonary vascular resistance [PVR = (PAPm - PAOP)/blood flow to each lung, where blood flow was the % of perfusion reaching each lung multiplied by CO] and regional pulmonary arterial compliance (i.e. the stroke volume to each lung divided by PA pulse pressure) [PA compliance = (blood flow to each lung/HR)/(PAPs-PAPd)].

*Respiratory mechanics (lung stress):* at each timepoint we collected measurements of airway peak pressure (Ppeak), plateau pressure (Pplat) by a 3-second inspiratory pause, mean airway pressure (Pmean), total PEEP (PEEPtot) by a 3-second expiratory pause, Vt, change between inspiratory and expiratory esophageal pressure. From these, driving pressure (DP) was calculated as Pplat –PEEPtot and driving transpulmonary pressure as DP- ΔPes; static respiratory system compliance (CRS) was calculated as VT/DP; lung compliance (CL) as VT/ΔPL; chest wall elastance (CCW) as VT/ΔPes.

*Bronchoalveolar lavage (BAL):* right after T24 measurements, a bilateral BAL was performed with 30 ml 0.9% saline solution. BAL fluids were then centrifugated. The supernatants were assayed by ELISA for concentration of regional inflammatory mediators (IL-6 and IL-1β).

*Euthanasia, autopsy, histology samples.* At the end of the experiment, animals were euthanized (by intravenous injection of potassium chloride 40 mEq under deep sedation) and underwent autopsy for collection of histological samples. Lungs were promptly excised en bloc via sternotomy and the correct position of the endobronchial tube was confirmed by direct visualization. For each pig’s lungs six representative tissue samples (2 from upper, 2 from medial and 2 from inferior lobes for each side) of 1 cm3 volume were collected and stored in formaldehyde to undergo histological examination. Two pathologists blinded to study group and to the original location of the sample conducted the examination. Right and left histological score of the lungs was calculated from the six samples per animal stored in formaldehyde (3 for each side). Ten main histological alterations were evaluated: emphysematous change, interstitial congestion, alveolar hemorrhage, alveolar neutrophil infiltration, alveolar macrophage proliferation, alveolar type II pneumocytes proliferation, interstitial lymphocytes proliferation, interstitial thickening, hyaline membrane formation and organization of alveolar exudate. Each alteration was scored from 0 (absent) to 3 (severe) in each sample (ten values from zero to 3 for each sample); then, the histological score of that sample was calculated as the sum of the ten values; finally, the regional histological score of the lungs was calculated as the average value of the scores of the 3 samples for each side (range: 0 to 30).

**Right lung injury**

The right lung of both groups was hyperperfused (Figure 4), with the right side of the NPLI group receiving 100% of the cardiac output trough the pulmonary artery vs. around 80% for NVLI.

In terms of V’/Q mismatch, the right lung of the NVLI group was characterized by ventilation to either dead space (40%) or high V’/Q (60%) compartments (Online Figure 4). This likely generated regional hypocapnia. The NVLI group, instead, was characterized by larger fraction of ventilation and perfusion reaching the normal V’/Q compartment of the right lung (Online Figure 6).

Values of pulmonary artery pressure were higher in the NVLI vs. NPLI group (Figure 8), increasing the risk of vascular leak and lung edema in the right well perfused lung.

The NVLI group was characterized by higher values of dynamic and static lung stress to the right ventilated side (Figure 9), as compared to the NPLI group.

Opposite to what we observed in the left compartments, right lung from NVLI and NPLI groups showed different proteomic profiles at unsupervised analysis (Figure 10). Significantly enriched proteins in the NVLI right lung (Figure 10) included the IL5, a driver of eosinophil-driven inflammation during severe asthma [5], as well as factors involved in respiratory virus infection responses and host interactions, as evidenced by pathway analysis (Figure 10 and Figure Online 2). Specifically, we found overexpression in the right NVLI lung of the *coxsackievirus and adenovirus receptor* (CXADR), which is involved in transepithelial migration of leukocytes, the *leukocyte immunoglobulin-like receptor subfamily B member 4* (LLRB4), the *antiviral innate immune response receptor RIG-I* (DDX58), the *Fc receptor-like protein 3* (FCRL3), which promotes TLR9-induced B-cell proliferation, the *Islet Cell Autoantigen 1* (ICA1) the *Aryl Hydrocarbon Receptor Nuclear Translocator* (ARNT) and the *diacylglycerol kinase Z* (DGKZ), which is involved in platelet activation. In the right NPLI lung, we found overexpression of the transcription suppressor BACH1, a crucial sensor of cell stress that regulates ROS production and immunity [6] also in the context of ischemia-induced angiogenesis in endothelial cells [7-8], of GLB1 and of the *Phosphoinositide 3-kinase adapter protein 1* (PIK3AP1). Upregulation of BACH1 and of GLB1 in the right lung was also detected when we compared the right and left organs within the NPLI group (Figure Online 3B).

**Control group**

Animals of the Control group (n=6) were ventilated for 24 hours with the same ventilatory settings as the other 2 groups (NVLI and NPLI): Volume-controlled mode, Vt 15 ml/kg, PEEP 1 cmH_2_O, respiratory rate 15 bpm, I:E 1:2 and FiO2 0.5.

In the Supplementary Table 2 are shown the histological and some physiological and EIT variables obtained at the end of experiment (T24).

**Rerences**

1. Spinelli E, Perez J, Chiavieri V, Leali M, Mansour N, Madotto F, Rosso L, Panigada M, Grasselli G, Vaira V, Mauri T. Pathophysiological Markers of Acute Respiratory Distress Syndrome Severity Are Correlated With Ventilation-Perfusion Mismatch Measured by Electrical Impedance Tomography. Crit Care Med 2024; doi: 10.1097/CCM.0000000000006458
2. Pulletz S, Elke G, Zick G, Schädler D, Scholz J, Weiler N, Frerichs I. Performance of electrical impedance tomography in detecting regional tidal volumes during one-lung ventilation. Acta Anaesthesiol Scand 2008;52(8):1131-9
3. He H, Chi Y, Long Y, Yuan S, Zhang R, Yang Y, Frerichs I, Möller K, Fu F, Zhao Z. Three broad classifications of acute respiratory failure etiologies based on regional ventilation and perfusion by electrical impedance tomography: a hypothesis-generating study. Ann Intensive Care 2021;11(1):134
4. Pavlovsky B, Pesenti A, Spinelli E, Scaramuzzo G, Marongiu I, Tagliabue P, Spadaro S, Grasselli G, Mercat A, Mauri T. Effects of PEEP on regional ventilation-perfusion mismatch in the acute respiratory distress syndrome. Crit Care 2022;26(1):211
5. Bajbouj K, AbuJabal R, Sahnoon L, Olivenstein R, Mahboub B, Hamid Q. IL-5 receptor expression in lung fibroblasts: Potential role in airway remodeling in asthma. *Allergy*. 2023;78(3):882-885. doi:10.1111/all.15627
6. Zhang X, Guo J, Wei X, et al. Bach1: Function, Regulation, and Involvement in Disease. Oxid Med Cell Longev. 2018;2018:1347969. Published 2018 Oct 2. doi:10.1155/2018/1347969
7. Yusoff FM, Maruhashi T, Kawano KI, et al. Bach1 plays an important role in angiogenesis through regulation of oxidative stress. Microvasc Res. 2021;134:104126. doi:10.1016/j.mvr.2020.104126
8. Jia M, Li Q, Guo J, et al. Deletion of BACH1 Attenuates Atherosclerosis by Reducing Endothelial Inflammation. Circ Res. 2022;130(7):1038-1055. doi:10.1161/CIRCRESAHA.121.319540

**Supplementary Table 1. Physiological variables along the experiment in the NVLI and NPLI groups**

|  | **T2** | **T6** | **T12** | **T18** | **T24** | **Time** | **Group** | **Time*Group** |
| --- | --- | --- | --- | --- | --- | --- | --- | --- |
| **Respiratory mechanics** | | | | | | | | |
| **Peak Pressure (cmH_2_O)** |  | | | | | | | |
| NVLI | 33 ± 5* | 34 ± 6* | 38 ± 11 | 34 ± 6 | 36 ± 9 | **<0.001** | **0.003** | 0.275 |
| NPLI | 25 ± 4 | 27 ± 3 | 27 ± 2 | 30 ± 4 | 33 ± 7 |  |  |  |
| **Mean Airway Pressure (cmH_2_O)** |  | | | | | | | |
| NVLI | 8 ± 1* | 8 ± 1 | 10 ± 2 | 11 ± 6 | 10 ± 3 | **0.008** | **0.007** | 0.272 |
| NPLI | 7 ± 1 | 7 ± 1 | 8 ± 1 | 8 ± 1 | 9 ± 2 |  |  |  |
| **Respiratory System Compliance (ml/cmH_2_O)** |  | | | | | | | |
| NVLI | 22 ± 2* | 22 ± 3* | 20 ± 4* | 21 ± 4* | 20 ± 4* | <0.001 | <0.001 | 0.010 |
| NPLI | 34 ± 8 | 31 ± 5 | 30 ± 3 | 28 ± 4 | 25 ± 4 |  |  |  |
| **Lung Compliance (ml/cmH_2_O)** |  | | | | | | | |
| NVLI | 28 ± 5* | 28 ± 6* | 25 ± 7* | 29 ± 10 | 26 ± 12 | **0.009** | **<0.001** | 0.316 |
| NPLI | 44 ± 13 | 43 ± 12 | 41 ± 9 | 38 ± 8 | 35 ± 11 |  |  |  |
| **Chest Wall Compliance (ml/cmH_2_O)** |  | | | | | | | |
| NVLI | 128 ± 55 | 126 ± 64 | 139 ± 83 | 105 ± 45 | 97 ± 31 | 0.370 | 0.278 | 0.593 |
| NPLI | 207 ± 165 | 203 ± 274 | 141 ± 65 | 132 ± 47 | 114 ± 35 |  |  |  |
| **Gas Exchange** | | | | | | | | |
| **PaCO_2_ (mmHg)** |  | | | | | | | |
| NVLI | 34 ± 3* | 33 ± 4 | 35 ± 10 | 30 ± 3 | 33 ± 7 | **0.003** | 0.701 | **<0.001** |
| NPLI | 44 ± 9 | 35 ± 6 | 31 ± 5 | 33 ± 15 | 29 ± 5 |  |  |  |
| **pH** |  | | | | | | | |
| NVLI | 7.55 ± 0.03* | 7.55 ± 0.04 | 7.50 ± 0.09 | 7.56 ± 0.03 | 7.50 ± 0.09 | 0.106 | 0.972 | **<0.001** |
| NPLI | 7.46 ± 0.06 | 7.53 ± 0.05 | 7.56 ± 0.05 | 7.56 ± 0.05 | 7.54 ± 0.02 |  |  |  |
| **HCO_3_^-^** |  | | | | | | | |
| NVLI | 30 ± 2 | 29 ± 2 | 27 ± 2 | 24 ± 4 | 25 ± 2 | **0.037** | 0.107 | 0.119 |
| NPLI | 30 ± 1 | 29 ± 2 | 29 ± 3 | 30 ± 10 | 26 ± 3 |  |  |  |
| **EtCO_2_ (mmHg)** |  | | | | | | | |
| NVLI | 30 ± 3 | 30 ± 3 | 29 ± 3 | 27 ± 3 | 28 ± 3 | **<0.001** | 0.094 | 0.063 |
| NPLI | 30 ± 6 | 28 ± 3 | 26 ± 3 | 24 ± 4 | 25 ± 3 |  |  |  |
| **Haemodynamics** | | | | | | | | |
| **Systolic Arterial Pressure (mmHg)** |  | | | | | | | |
| NVLI | 122 ± 13 | 121 ± 15 | 121 ± 12 | 117 ± 13 | 111 ± 6 | **<0.001** | 0.639 | 0.303 |
| NPLI | 128 ± 10 | 122 ± 10 | 118 ± 11 | 109 ± 12 | 106 ± 8 |  |  |  |
| **Diastolic Arterial Pressure (mmHg)** |  | | | | | | | |
| NVLI | 91 ± 15 | 88 ± 18 | 85 ± 17 | 80 ± 14 | 71 ± 6 | **<0.001** | 0.930 | 0.745 |
| NPLI | 96 ± 11 | 88 ± 14 | 84 ± 13 | 72 ± 13 | 70 ± 8 |  |  |  |
| **Mean Arterial Pressure (mmHg)** |  | | | | | | | |
| NVLI | 106 ± 16 | 103 ± 18 | 91 ± 32 | 99 ± 14 | 90 ± 8 | **0.003** | 0.551 | 0.549 |
| NPLI | 112 ± 11 | 105 ± 13 | 101 ± 11 | 91 ± 14 | 88 ± 9 |  |  |  |
| **Central Venous Pressure (mmHg)** |  | | | | | | | |
| NVLI | 6 [5 – 9] | 6 [5 – 8] | 6 [5 – 9] | 6 [6 – 8] | 7 [5 – 9] | 0.267 | **0.008** | 0.852 |
| NPLI | 4 [4 – 4] | 4 [ 3 – 5] | 4 [3 – 4] | 4 [3 – 5] | 4 [3 – 5] |  |  |  |
| **Pulmonary Capillary Wedge Pressure (mmHg)** |  | | | | | | | |
| NVLI | 8 ± 3 | 8 ± 3 | 7 ± 3 | 8 ± 3 | 9 ± 3 | 0.474 | **0.003** | 0.097 |
| NPLI | 5 ± 2 | 4 ± 1 | 5 ± 3 | 4 ± 2 | 4 ± 2 |  |  |  |
| **Heart Rate (bpm)** |  | | | | | | | |
| NVLI | 106 ± 12 | 110 ± 13 | 107 ± 13 | 103 ± 10 | 102 ± 17 | **0.037** | 0.164 | 0.972 |
| NPLI | 99 ± 15 | 102 ± 16 | 99 ± 11 | 96 ± 15 | 93 ± 11 |  |  |  |
| **SvO_2_ (%)** |  | | | | | | | |
| NVLI | 54 ± 7 | 49 ± 7 | 43 ± 9* | 52 ± 8 | 40 ± 21 | **0.005** | **0.006** | 0.097 |
| NPLI | 59 ± 5 | 55 ± 4 | 54 ± 5 | 54 ±5 | 52 ± 9 |  |  |  |
| **Lactate (mmol/L)** |  | | | | | | | |
| NVLI | 1.0 [0.8 – 1.2] | 0.8 [0.7 – 1.1] | 0.8 [0.7 – 0.9] | 0.7 [0.6 – 0.8] | 0.7 [0.6 – 0.8] | **<0.001** | 0.135 | 0.365 |
| NPLI | 0.9 [0.6 – 1.1] | 0.6 [0.5 – 0.7] | 0.7 [0.6 – 0.7] | 0.7 [0.6 – 0.8] | 0.7 [0.6 – 0.7] |  |  |  |
| **Others** | | | | | | | | |
| **Fluid Balance** |  | | | | | | | |
| NVLI | -42 ± 117* | 37 ± 142 | 182 ± 165 | 147 ± 31 | 155 ± 195 | **<0.001** | 0.065 | 0.365 |
| NPLI | 185 ± 123 | 165 ± 207 | 252 ± 258 | 327 ± 352 | 397 ± 312 |  |  |  |

Data are expressed as mean ± SD or median [interquartile range], as appropriate. Statistical analysis was performed using mixed-effect model for repeated measurements, including group and time as main independent factors and group-by-time interaction, and followed by Sidak’s multiple comparison test.

* p<0.05 vs NPLI group.

*Abbreviations: PaCO_2_: partial pressure of carbon dioxide; EtCO_2_: end-tidal CO_2_; SvO_2_: mixed venous blood oxygen saturation.*

**Supplementary Table 2. Histological and physiological variables at the end of experiment in the Control group (n=6).**

|  | **T24** |
| --- | --- |
| **Histological score** | |
| **Left lung** | 2 ± 1 |
| **Right lung** | 3 ± 1 |
| **Respiratory mechanics** | |
| **Driving Pressure (cmH_2_O)** | 15 ± 3 |
| **Transpulmonary Pressure (cmH_2_O)** | 13 ± 4 |
| **Respiratory System Compliance (ml/cmH_2_O)** | 33 ± 4 |
| **Gas exchange** | |
| **PaCO_2_ (mmHg)** | 29 ± 4 |
| **PaO_2_/FiO_2_** | 406 ± 49 |
| **Hemodynamics** | |
| **Mean Arterial Pressure (mmHg)** | 94 ± 11 |
| **Mean Pulmonary Artery Pressure (mmHg)** | 19 ± 4 |
| **EIT data** | |
| **Left lung ventilation (%)** | 50 ± 9 |
| **Left lung perfusion (%)** | 51 ± 11 |

Data are expressed as mean ± SD.

*Abbreviations: PaCO_2_: partial pressure of carbon dioxide; PaO_2_/FiO_2_: arterial partial pressure of oxygen/fraction of inspired oxygen; EIT: Electrical Impedance Tomography.*

**Figure Online 1. Representative images of the lungs at the end of the experiment.**

*NVLI: Non-Ventilated Lung Injury; NPLI: Non-Perfused Lung Injury.*

**Figure Online 2.** Pathway analysis was performed in Reactome using the overrepresentation function for proteins upregulated in NVLI left and right lung compared with NPLI. *NVLI: Non-Ventilated Lung Injury; NPLI: Non-Perfused Lung Injury.*


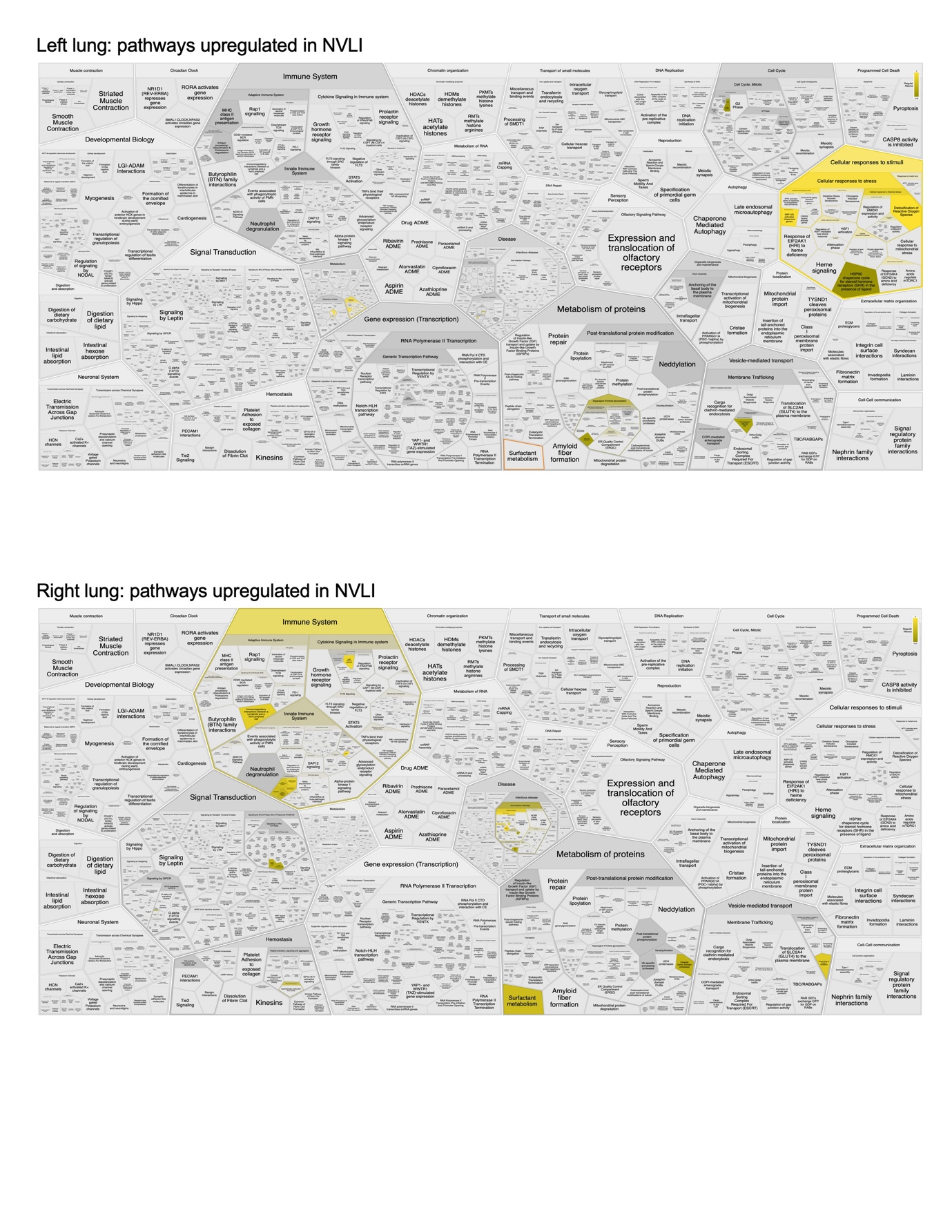


**Figure Online 3. The Immune Response proteome in NVLI and NPLI groups.** The supervised heatmaps show protein expression in right and left lung samples. Significantly different proteins in the right (RL) and left (LL) lung from NVLI (A) and NPLI (B) groups are reported in the tables along with the mean expression value of and p-value. *NVLI: Non-Ventilated Lung Injury; NPLI: Non-Perfused Lung Injury.*

**
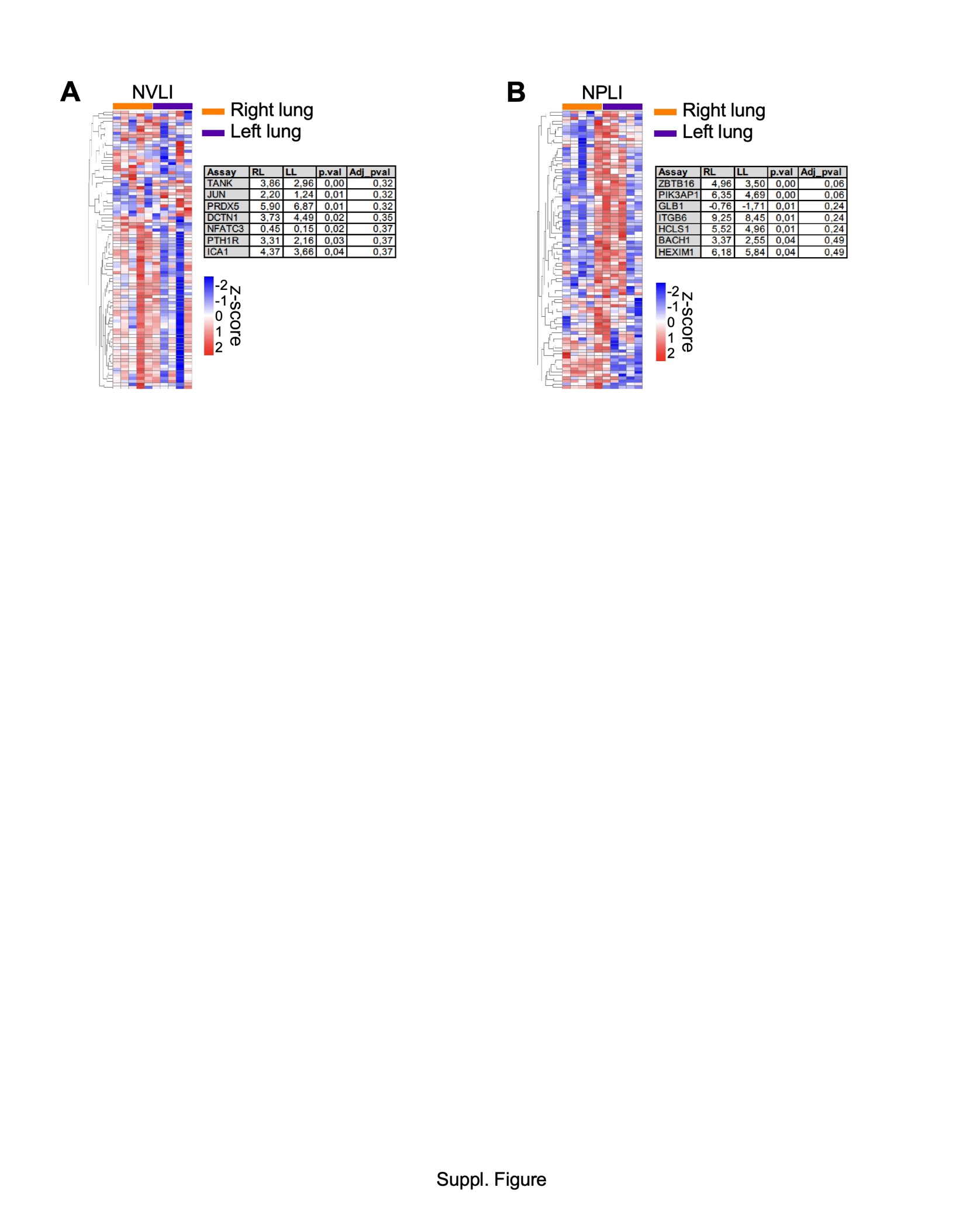
**

**Figure Online 4. Right lung histological injury score sub-items.**

The right lung of the NVLI group showed more alveolar hemorrhage (A), alveolar macrophage proliferation (B) and hyaline membrane formation (C) compared to the right lung of the NPLI group.

Data are expressed as scatter plot with bars and error bars (mean ± SEM). Statistical analysis is performed by unpaired t-test or Mann-Whitney U test, as appropriated, p-values are reported in the graphs. *p<0.05, ***p<0.001, ****p<0.0001. *RL, right lung; NVLI, non-ventilated lung injury; NPLI, non-perfused lung injury.*

**Figure Online 5. Cytokines concentration from the right BAL fluid.** Alveolar concentration of IL-1β and IL-6 was measured in the right lung (A and B, respectively) of each study group.

Data are expressed as scatter plot with bars (mean ± SEM) where each sample is a dot. Statistical analysis is performed by unpaired t-test or Mann-Whitney U test, as appropriated. *p<0.05, ***p<0.001, ****p<0.0001. *NVLI, non-ventilated lung injury; NPLI, non-perfused lung injury.*

**Figure Online 6. EIT analysis of distribution of ventilation and perfusion to the different V’/Q compartments in the right lung.** Panel 1: EIT-measured fraction of ventilation reaching the dead space (A), high V’/Q (B) normal V’/Q (C) and low V’/Q (D) units in the right lung. In the NVLI group the ventilation reached mainly dead space and high V’/Q compartments, while in the NPLI group the ventilation of the normal V’/Q compartment was higher. Panel 2: EIT-measured fraction of perfusion reaching the high V’/Q (A), normal V’/Q (B), low V’/Q (C) and shunt (D) units in the right lung. The right lung of the NVLI group was highly unmatched with around 80% of perfusion distributing to high V’/Q compartment, while in the NPLI group the perfusion reached more homogeneously high and normal V’/Q compartments.

Data are expressed as mean ± SEM. Comparisons are obtained with mixed-effect model for repeated measures followed by Sidak’s post-hoc test with groups and time as independent factors and group x time interaction. P-values are reported in the graphs. *NVLI, non-ventilated lung injury; NPLI, non-perfused lung injury; V: ventilation; P: perfusion; V/Q ventilation to perfusion.*
